# Construction and external validation of an early warning model for piperacillin/tazobactam-associated hypokalemia in critically ill patients

**DOI:** 10.3389/fphar.2026.1886455

**Published:** 2026-08-24

**Authors:** Yuan-Min Wang, Fu-Kun Zhao, Cang-Hong Ou-Yang, Rong-Ping Zhu, Min Luo

**Affiliations:** 1 The Third Affiliated Hospital of Zunyi Medical University, The First People’s Hospital of Zunyi, Clinical Pharmacy Department, Zunyi, Guizhou, China; 2 School of Pharmacy, Zunyi Medical University, Zunyi, Guizhou, China

**Keywords:** critical care, hypokalemia, machine learning, MIMIC-IV database, piperacillin/tazobactam, risk prediction

## Abstract

**Background:**

Hypokalemia is a common adverse event during piperacillin/tazobactam (TZP) therapy in critically ill patients. This study developed and externally validated machine learning models to predict hypokalemia using baseline variables.

**Methods:**

Data were obtained from the Medical Information Mart for Intensive Care (MIMIC)-IV database and an external cohort from the Third Affiliated Hospital of Zunyi Medical University. Hypokalemia was defined as the first serum potassium <3.5 mmol/L within 14 days after TZP initiation. Variables with >20% missing data were excluded; remaining missing values were imputed. Least Absolute Shrinkage and Selection Operator (LASSO) regression with 10-fold cross-validation based on binomial deviance was used for feature selection. Logistic Regression, Random Forest, Extreme Gradient Boosting Model (XGBoost), Adaptive Boosting (AdaBoost), and Gradient Boosting Machine models were developed. Performance was assessed with receiver operating characteristic curves, the area under the receiver operating characteristic curve (AUC), the area under the precision-recall curve (AUPRC), calibration, precision-recall analysis, and decision curve analysis.

**Results:**

The internal cohort included 1,345 patients (594 hypokalemia), and the external cohort included 421 patients (159 hypokalemia). Internal AUCs were 0.759 (Random Forest), 0.738 (GBM), 0.735 (XGBoost), 0.716 (AdaBoost), and 0.694 (Logistic Regression). External AUCs were 0.668 (GBM), 0.654 (XGBoost), 0.648 (Random Forest), 0.645 (AdaBoost), and 0.620 (Logistic Regression). The Random Forest model achieved an AUPRC of 0.701 in the internal cohort, while GBM achieved the highest AUPRC in the external cohort (0.554). Calibration and decision curve analyses demonstrated acceptable agreement and potential clinical utility. SHapley Additive exPlanations (SHAP) analysis identified baseline potassium, potassium-wasting diuretic use, serum creatinine, age, renal disease, serum sodium, and albumin as the most influential predictors of TZP-associated hypokalemia.

**Conclusion:**

Machine learning models achieved moderate predictive performance. Baseline potassium and potassium-wasting diuretic use were the strongest predictors.

## 1 Introduction

Piperacillin/tazobactam (TZP), a combination of a broad-spectrum semisynthetic *β*-lactam antibiotic and a *β*-lactamase inhibitor, is widely used in clinical practice and remains one of the most frequently prescribed antimicrobial agents in intensive care units (ICUs) (Tomazini et al., 2024). It demonstrates potent activity against Gram-negative pathogens, including extended-spectrum *β*-lactamase-producing Enterobacteriaceae and certain strains of *Pseudomonas aeruginosa* (Islam et al., 2022). Despite its broad clinical utility, TZP administration has been associated with several adverse events, including cutaneous hypersensitivity reactions, leukopenia, gastrointestinal disturbances, acute kidney injury, and hypokalemia (Wang et al., 2020; Pandya et al., 2024; Casimir-Brown et al., 2021; Seo and Kim, 2023). Among these, hypokalemia has attracted increasing clinical attention in recent years. In November 2020, Japanese regulatory authorities issued a safety warning regarding hypokalemia occurring during TZP therapy and required the prescribing information for TZP injection to be updated to include hypokalemia under “clinically significant adverse reactions” (Cao et al., 2023; Gogo-Peters et al., 2026). Pharmacovigilance Programme of India (PvPI) has also issued a drug alert that TZP may induce hypokalemia (Wan et al., 2026).

Previous studies have reported substantial variability in the incidence of hypokalemia during TZP therapy across different infectious disease settings (Tomazini et al., 2024). For example, the reported incidence was approximately 1.5% among patients with urinary tract infections (Seo and Kim, 2022), whereas studies involving hospital-acquired pneumonia reported incidences ranging from <1% to 24.8% (Seo and Kim, 2023). Critically ill patients are particularly susceptible to electrolyte disturbances because of severe physiological derangements, organ dysfunction, intensive monitoring, and exposure to multiple medications. In this setting, hypokalemia occurring during TZP therapy may be more frequently observed and may require closer clinical attention (Seo and Kim, 2023; Devaki et al., 2024). Failure to recognize and correct hypokalemia promptly may result in serious adverse consequences, including cardiac arrhythmias and neuromuscular dysfunction.

Electrolytes are essential for maintaining normal physiological function. As the principal intracellular cation, potassium is predominantly distributed within the intracellular compartment and plays a central role in cellular metabolism, intracellular osmotic regulation, acid-base balance, neuromuscular excitability, and maintenance of normal myocardial electrical activity (Stone et al., 2016; Jansen et al., 2024). Hypokalemia is one of the most common electrolyte abnormalities encountered in clinical practice and is generally defined as a serum potassium concentration <3.5 mmol/L (Brown and Paloian, 2023; Lin et al., 2023; Coregliano-Ring et al., 2022). Mild hypokalemia is frequently asymptomatic, whereas severe hypokalemia may cause respiratory depression, paralytic ileus, and life-threatening ventricular arrhythmias. In critically ill patients, these manifestations may be overlooked or masked by underlying disease processes, making early recognition more challenging. Drug-related hypokalemia is particularly difficult to identify because electrolyte abnormalities may result from multiple concurrent clinical factors. Therefore, identifying patients at increased risk of hypokalemia during TZP therapy and developing reliable predictive models may facilitate early monitoring and individualized clinical management (Seo and Kim, 2022; Lin et al., 2023).

In the present study, we conducted a retrospective analysis of critically ill patients who received TZP using data extracted from the MIMIC-IV database. Demographic and clinical variables available at treatment initiation were collected, including sex, age, infection site, baseline serum potassium level, laboratory findings, and comorbidities. The objectives of this study were to identify baseline predictors associated with hypokalemia occurring during TZP therapy in critically ill patients, to develop machine learning-based prediction models, and to perform both internal and external validation. Our findings may provide a useful reference for early risk stratification and safer clinical monitoring during TZP treatment.

## 2 Materials and methods

### 2.1 Materials

#### 2.1.1 Data sources

This retrospective study used data from the MIMIC-IV database (version 2.0) and the PASS system of the Third Affiliated Hospital of Zunyi Medical University. The publicly available MIMIC-IV database was jointly developed by Beth Israel Deaconess Medical Center (BIDMC) and the Massachusetts Institute of Technology (MIT). Access is granted to qualified researchers who have completed human subjects research training and signed a data use agreement. The database contains de-identified clinical data from critically ill patients admitted between 2008 and 2019 (Johnson et al., 2023). The investigators were authorized to access the database (Record ID: 67044571).

#### 2.1.2 Inclusion and exclusion criteria

The inclusion criteria were as follows: ① age ≥18 years, regardless of sex; ② treatment with TZP with at least one serum potassium measurement before or at treatment initiation; ③ a last pre-treatment serum potassium level of ≥3.5 mmol/L.

The study endpoint of hypokalemia was defined as the first serum potassium measurement <3.5 mmol/L occurring within 14 days after TZP initiation, ensuring a clinically relevant observation window for event occurrence. To reduce potential bias, variables observed only during treatment, such as treatment duration (DUD), were excluded from baseline predictors to prevent data leakage.

The exclusion criteria were as follows: ① receipt of peritoneal dialysis or hemodialysis; ② incomplete clinical data; and ③ diseases or clinical conditions known to affect potassium homeostasis, including alkalosis, vomiting, diarrhea, tube drainage, hypomagnesemia, hypokalemic thyrotoxic periodic paralysis, primary hyperaldosteronism, Cushing syndrome, and renal tubular acidosis. The study protocol was approved by the Ethics Committee of the First People’s Hospital of Zunyi (Approval No [2025]-1-323).

### 2.2 Missing data handling

Missing data for all candidate variables were systematically evaluated before model development. Variables with missing rates greater than 20 percent were excluded from further analysis to reduce potential bias and instability. The proportion of missingness for each variable is summarized in Supplementary Table S1.

For the remaining variables, missing data were handled using multiple imputation by chained equations (MICE). Continuous variables were imputed using predictive mean matching, whereas categorical variables were imputed using logistic regression models.

Multiple imputation was performed to reduce bias introduced by missing data and to preserve the underlying distribution of the dataset. For subsequent machine learning model development, a single completed dataset derived from the imputation procedure was used to ensure consistency across different algorithms and to maintain computational stability during model training and validation.

After imputation, the completed dataset was checked for data completeness and variable consistency before subsequent model development.

### 2.3 Feature selection

For baseline risk prediction, all candidate variables available at the initiation of TZP therapy were included in the feature selection process using LASSO regression with a binomial model (Li et al., 2025). Continuous variables were standardized, and categorical variables were encoded appropriately prior to analysis. The optimal regularization parameter (lambda) was selected based on the minimum binomial deviance in 10-fold cross-validation for the LASSO logistic regression model. Variables with non-zero coefficients at the selected lambda were retained for model development. All machine learning models were developed based on the single imputed dataset.

### 2.4 Model development and validation

Data extracted from the MIMIC-IV database (version 2.0) were randomly divided into a training set and a test set at a ratio of 8.5:1.5 using stratified sampling to preserve the proportion of hypokalemia events.

A Random Forest model was developed as the primary prediction model. The final Random Forest model was constructed using 300 trees (ntree = 300), with the number of variables randomly selected at each split determined as the square root of the total number of predictors (mtry = √p). The minimum terminal node size was set to 30, and the maximum number of terminal nodes was limited to 15.

For comparative evaluation, Logistic Regression, AdaBoost, XGBoost, Random Forest, and GBM models were additionally developed using the same training and testing datasets (Zhang et al., 2025). Model discrimination was assessed using receiver operating characteristic (ROC) curves and the area under the receiver operating characteristic curve (AUC). Calibration performance was evaluated using calibration curves. Predictive precision was assessed using precision-recall (PR) curves and the area under the precision-recall curve (AUPRC), and clinical utility was evaluated using decision curve analysis (DCA).

For all performance metrics, the optimal classification threshold was determined using the Youden index. Confidence intervals for AUC were calculated using DeLong’s method, whereas 95% confidence intervals for Accuracy, Sensitivity, Specificity, Positive Predictive Value (PPV), Negative Predictive Value (NPV), F1 score, AUPRC, and Brier score were estimated using 1,000 bootstrap resamples.

For external validation, 421 patients admitted between January 2020 and December 2024 to the ICU, neurological intensive care unit (NICU), and respiratory ICU of the Third Affiliated Hospital of Zunyi Medical University were included. The same evaluation framework was applied to the external validation cohort to assess model generalizability and robustness.

## 3 Results

### 3.1 Baseline characteristics

**FIGURE 1:**
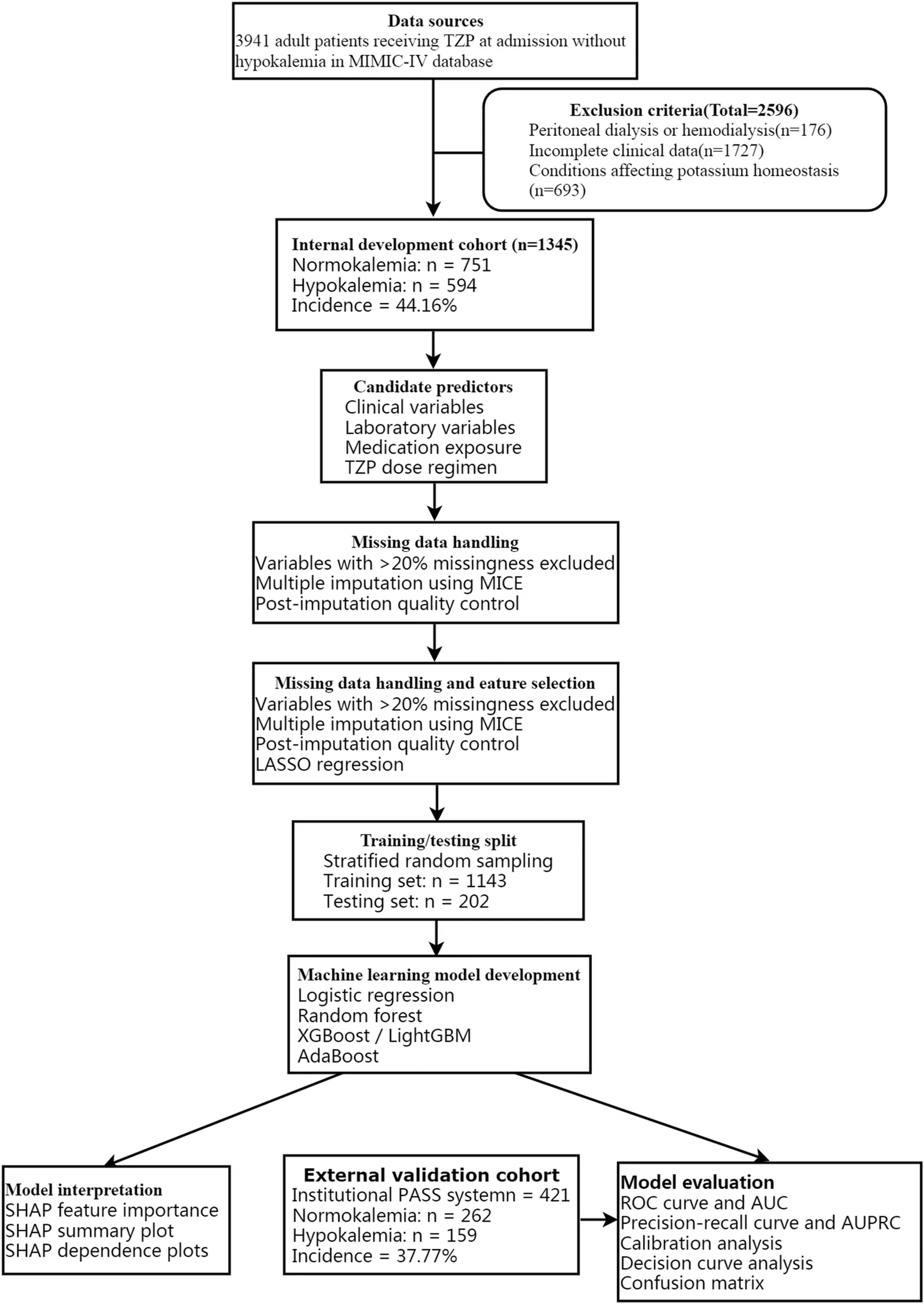
Study workflow for cohort selection, feature processing, model development, and external validation.

The study workflow is shown in Figure 1. After eligibility screening, 1,345 patients from the MIMIC-IV database were included in the internal development cohort. Among them, 751 patients remained normokalemic and 594 developed hypokalemia, corresponding to an incidence of 44.16%. The external validation cohort included 421 patients from the institutional PASS system, including 262 normokalemic patients and 159 patients with hypokalemia, with an incidence of 37.77%.

Baseline characteristics of the internal and external cohorts are presented in Table 1. In the internal cohort, patients who developed hypokalemia were older than normokalemic patients and had lower baseline potassium levels. The median baseline potassium concentration was 4.10 mmol/L in the hypokalemia group and 4.40 mmol/L in the normokalemia group. Differences were also observed in BMI, albumin, aspartate aminotransferase, alanine aminotransferase, total bilirubin, platelet count, serum sodium, serum creatinine, sex, diabetes, renal disease, potassium-wasting diuretic use, glucocorticoid use, TZP 4.5 g q8h regimen, lung infection, skin/soft tissue infection, and bone infection.

**TABLE 1:** Baseline characteristics of the internal and external cohorts.

Variable	Internal cohort overall (n = 1,345)	Normokalemia (n = 751)	Hypokalemia (n = 594)	*P* value	External cohort overall (n = 421)
Age, years	65.50 [54.60–74.80]	64.00 [53.65–74.05]	66.90 [56.45–76.20]	0.019	71.00 [59.00–80.00]
BMI, kg/m^2^	26.30 [22.30–31.40]	26.80 [23.00–32.70]	25.65 [21.70–30.28]	<0.001	21.23 [20.76–24.22]
Baseline potassium, mmol/L	4.20 [3.90–4.60]	4.40 [4.05–4.70]	4.10 [3.80–4.47]	<0.001	3.90 [3.70–4.30]
Albumin, g/dL	3.30 [2.80–3.80]	3.30 [2.85–3.80]	3.20 [2.70–3.70]	0.025	3.59 [3.23–3.93]
Alkaline phosphatase, U/L	97.00 [71.00–146.00]	98.00 [73.00–151.00]	93.00 [69.00–142.75]	0.174	91.00 [72.00–119.00]
Aspartate aminotransferase, U/L	29.00 [19.00–55.00]	27.00 [18.00–50.50]	31.00 [20.00–65.75]	0.001	26.00 [19.00–34.00]
Alanine aminotransferase, U/L	23.00 [14.00–45.00]	22.00 [14.00–42.00]	24.00 [14.00–51.75]	0.022	18.00 [12.00–29.00]
Total bilirubin, mg/dL	0.50 [0.30–1.00]	0.50 [0.30–0.90]	0.60 [0.40–1.10]	<0.001	0.70 [0.50–0.96]
Platelet count, ×10^9^/L	240.00 [162.00–336.00]	253.00 [174.00–350.00]	228.50 [150.25–314.75]	<0.001	200.00 [146.00–257.00]
Serum sodium, mmol/L	137.00 [134.00–140.00]	137.00 [134.00–139.00]	138.00 [135.00–141.00]	<0.001	137.70 [135.50–140.20]
Serum creatinine, mg/dL	1.00 [0.70–1.50]	1.00 [0.70–1.60]	0.90 [0.70–1.40]	<0.001	0.84 [0.67–1.14]
Female sex, n (%)	551 (41.0%)	279 (37.2%)	272 (45.8%)	0.002	257 (61.0%)
Diabetes, n (%)	495 (36.8%)	319 (42.5%)	176 (29.6%)	<0.001	76 (18.1%)
Renal disease, n (%)	332 (24.7%)	228 (30.4%)	104 (17.5%)	<0.001	104 (24.7%)
Liver disease, n (%)	171 (12.7%)	88 (11.7%)	83 (14.0%)	0.25	69 (16.4%)
Potassium-wasting diuretic use, n (%)	539 (40.1%)	247 (32.9%)	292 (49.2%)	<0.001	179 (42.5%)
Potassium-sparing diuretic use, n (%)	47 (3.5%)	26 (3.5%)	21 (3.5%)	1	66 (15.7%)
Glucocorticoid use, n (%)	263 (19.6%)	132 (17.6%)	131 (22.1%)	0.047	171 (40.6%)
Insulin use, n (%)	680 (50.6%)	391 (52.1%)	289 (48.7%)	0.235	59 (14.0%)
NSAID use, n (%)	1,195 (88.8%)	660 (87.9%)	535 (90.1%)	0.239	91 (21.6%)
TZP 2.25 g q8h, n (%)	133 (9.9%)	75 (10.0%)	58 (9.8%)	0.965	0 (0.0%)
TZP 4.5 g q8h, n (%)	1,037 (77.1%)	561 (74.7%)	476 (80.1%)	0.022	354 (84.1%)
TZP 4.5 g q6h, n (%)	3 (0.2%)	2 (0.3%)	1 (0.2%)	1	67 (15.9%)
Lung infection, n (%)	469 (34.9%)	210 (28.0%)	259 (43.6%)	<0.001	282 (67.0%)
Abdominal infection, n (%)	382 (28.4%)	208 (27.7%)	174 (29.3%)	0.559	37 (8.8%)
Urinary/renal infection, n (%)	259 (19.3%)	134 (17.8%)	125 (21.0%)	0.159	38 (9.0%)
Skin/soft tissue infection, n (%)	252 (18.7%)	175 (23.3%)	77 (13.0%)	<0.001	14 (3.3%)
Brain infection, n (%)	4 (0.3%)	3 (0.4%)	1 (0.2%)	0.635	6 (1.4%)
Bone infection, n (%)	109 (8.1%)	84 (11.2%)	25 (4.2%)	<0.001	2 (0.5%)

Table 1 baseline characteristics of internal (n = 1,345) and external (n = 421) cohorts. Continuous variables are presented as median [interquartile range, IQR] and categorical variables as n (%). *P* values indicate comparisons between normokalemia and hypokalemia groups in the internal cohort.

The external validation cohort showed differences in baseline characteristics compared with the internal cohort. Patients in the external cohort were older, had a lower BMI, and showed a lower baseline potassium level. The distribution of medication exposure and infection sites also differed between cohorts, reflecting clinical heterogeneity between the MIMIC-IV population and the institutional validation population.

### 3.2 Feature selection

**FIGURE 2:**
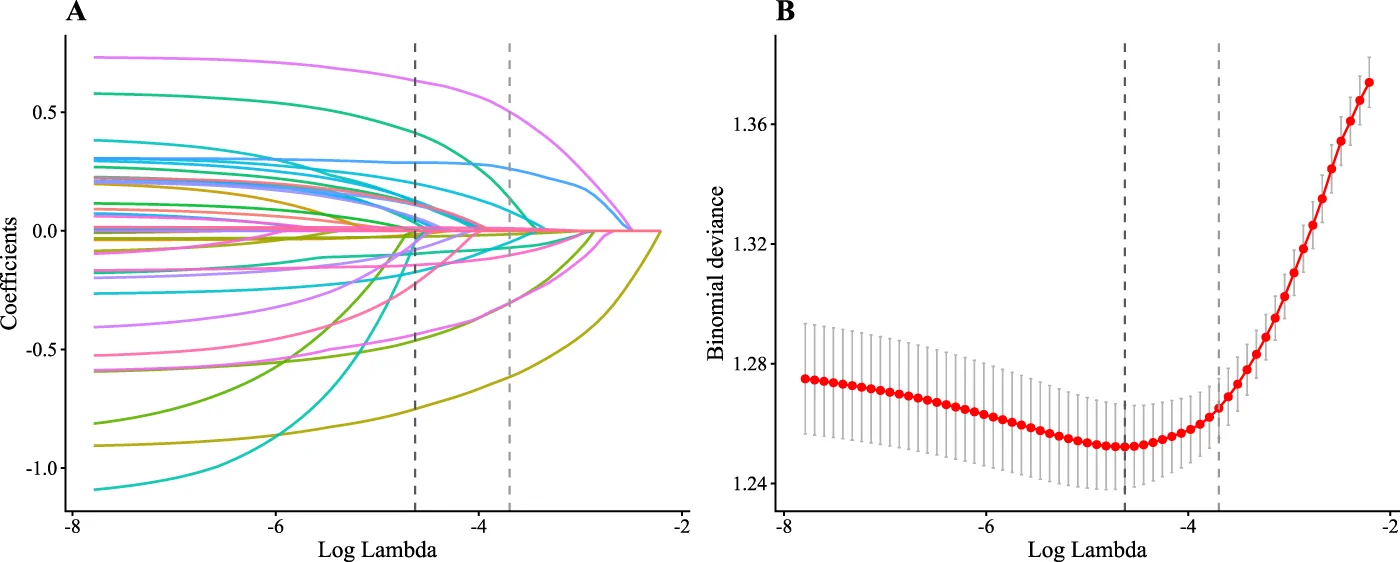
LASSO regression for feature selection. **(A)** Coefficient profiles of candidate variables. **(B)** Ten-fold cross-validation for selection of the optimal lambda based on binomial deviance.

LASSO regression was used to select predictors from the full candidate variable set available at TZP initiation. The coefficient paths of candidate variables are shown in Figure 2A. As the regularization parameter increased, coefficients of less informative variables gradually shrank toward zero.

The optimal lambda was determined using 10-fold cross-validation based on binomial deviance, as shown in Figure 2B. Variables with non-zero coefficients at the selected lambda were retained for subsequent model development. The final predictor set included baseline potassium, potassium-wasting diuretic use, renal disease, age, serum sodium, serum creatinine, albumin, female sex, and TZP 4.5 g q 8h regimen.

### 3.3 Model performance comparison

**FIGURE 3:**
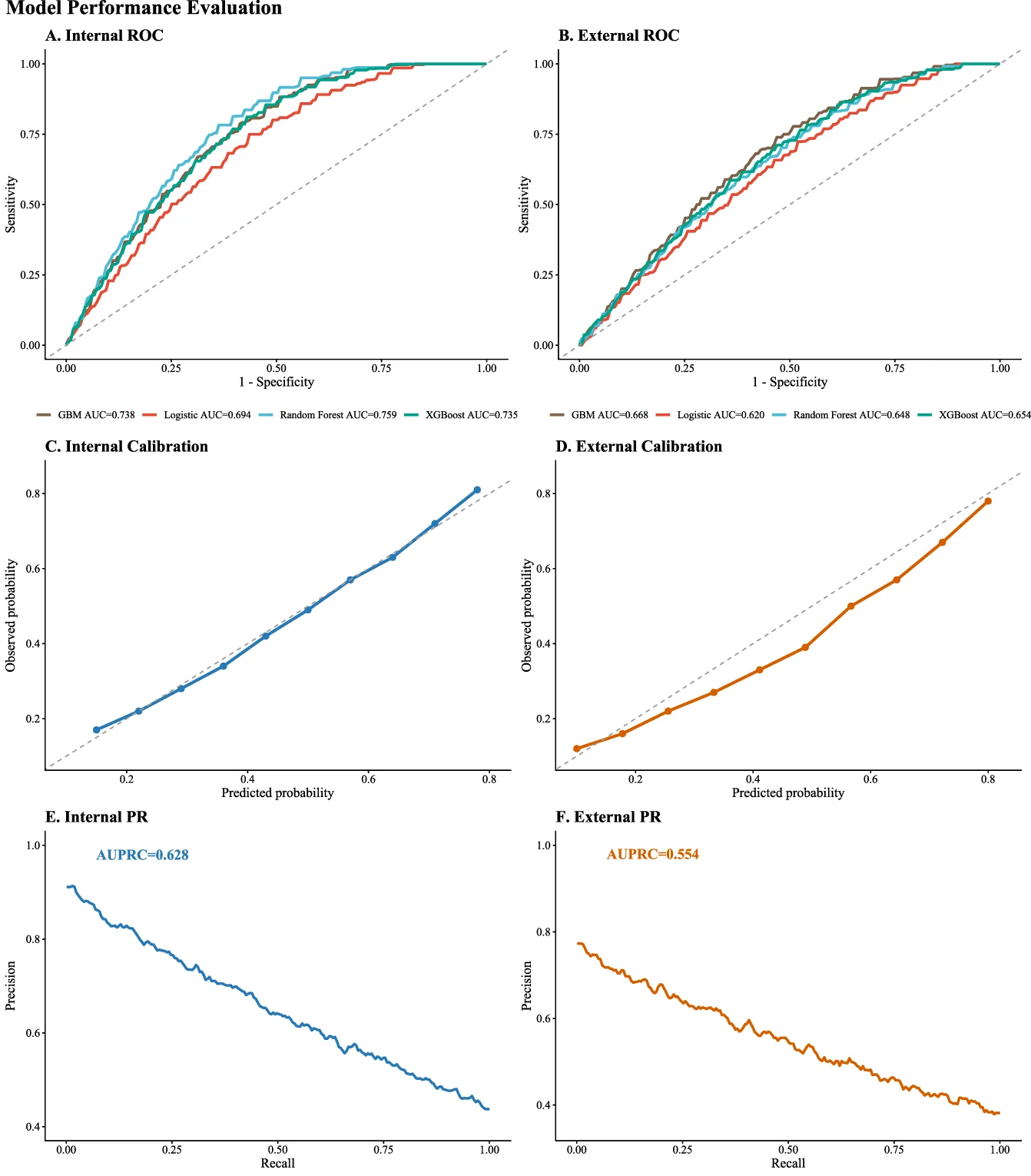
Model performance evaluation. **(A)** Internal ROC curves. **(B)** External ROC curves. **(C)** Internal calibration curve. **(D)** External calibration curve. **(E)** Internal precision-recall curve. **(F)** External precision-recall curve.

The predictive performance of five machine learning models, including Logistic Regression, Random Forest, AdaBoost, XGBoost, and GBM, was evaluated in the internal and external validation cohorts. Detailed performance metrics are summarized in Table 2, and the corresponding ROC, calibration, PR, and DCA results are presented in Figure 3.

**TABLE 2:** Performance comparison of machine learning models in the internal and external validation cohorts.

Cohort	Model	AUC (95% CI)	Optimal cutoff	Accuracy	Sensitivity	Specificity	PPV	NPV	F1 score	AUPRC	Brier score
Internal cohort	Logistic regression	0.694 (0.666–0.722)	0.417	0.642 (0.618–0.668)	0.712 (0.674–0.749)	0.586 (0.552–0.623)	0.576 (0.543–0.614)	0.720 (0.684–0.757)	0.637 (0.608–0.667)	0.628 (0.589–0.669)	0.219 (0.211–0.227)
Internal cohort	Random forest	0.759 (0.733–0.784)	0.388	0.697 (0.673–0.723)	0.705 (0.666–0.743)	0.691 (0.659–0.726)	0.644 (0.609–0.681)	0.748 (0.717–0.782)	0.673 (0.644–0.704)	0.701 (0.665–0.741)	0.207 (0.192–0.220)
Internal cohort	AdaBoost	0.716 (0.689–0.743)	0.386	0.654 (0.630–0.680)	0.734 (0.698–0.770)	0.591 (0.556–0.627)	0.587 (0.553–0.623)	0.738 (0.704–0.776)	0.652 (0.622–0.680)	0.648 (0.611–0.689)	0.213 (0.204–0.222)
Internal cohort	XGBoost	0.735 (0.708–0.761)	0.457	0.679 (0.652–0.705)	0.668 (0.629–0.708)	0.687 (0.654–0.721)	0.628 (0.591–0.667)	0.724 (0.691–0.758)	0.648 (0.616–0.679)	0.669 (0.631–0.709)	0.209 (0.201–0.216)
Internal cohort	GBM	0.738 (0.712–0.764)	0.426	0.676 (0.651–0.700)	0.724 (0.687–0.762)	0.638 (0.605–0.671)	0.613 (0.578–0.647)	0.745 (0.713–0.781)	0.664 (0.634–0.692)	0.677 (0.639–0.716)	0.208 (0.200–0.215)
External cohort	Logistic regression	0.620 (0.565–0.676)	0.582	0.625 (0.580–0.668)	0.503 (0.425–0.579)	0.698 (0.642–0.752)	0.503 (0.423–0.575)	0.698 (0.644–0.754)	0.503 (0.433–0.565)	0.516 (0.430–0.599)	0.249 (0.234–0.264)
External cohort	Random forest	0.648 (0.594–0.702)	0.635	0.629 (0.582–0.675)	0.579 (0.500–0.658)	0.660 (0.601–0.715)	0.508 (0.440–0.580)	0.721 (0.665–0.778)	0.541 (0.474–0.603)	0.519 (0.443–0.599)	0.272 (0.246–0.298)
External cohort	AdaBoost	0.645 (0.591–0.699)	0.53	0.622 (0.577–0.667)	0.629 (0.548–0.707)	0.618 (0.561–0.676)	0.500 (0.432–0.569)	0.733 (0.676–0.794)	0.557 (0.494–0.618)	0.533 (0.454–0.610)	0.242 (0.225–0.258)
External cohort	XGBoost	0.654 (0.600–0.708)	0.567	0.632 (0.589–0.677)	0.591 (0.513–0.675)	0.656 (0.599–0.714)	0.511 (0.445–0.585)	0.726 (0.667–0.784)	0.548 (0.482–0.610)	0.534 (0.457–0.618)	0.241 (0.227–0.254)
External cohort	GBM	0.668 (0.614–0.721)	0.574	0.648 (0.603–0.696)	0.597 (0.520–0.681)	0.679 (0.623–0.736)	0.531 (0.464–0.606)	0.736 (0.681–0.794)	0.562 (0.496–0.625)	0.554 (0.474–0.636)	0.238 (0.224–0.251)

Values are presented as point estimates with 95% confidence intervals, except for the optimal cutoff. The optimal cutoff value was determined using the Youden index. Confidence intervals for AUC, were calculated using DeLong’s method, and confidence intervals for all other performance metrics were estimated using 1,000 bootstrap resamples. AUC, area under the receiver operating characteristic curve; CI, confidence interval; PPV, positive predictive value; NPV, negative predictive value; AUPRC, area under the precision-recall curve.

In the internal cohort, Random Forest achieved the best discriminative performance, with an AUC of 0.759 (95% CI: 0.733–0.784), followed by GBM (AUC = 0.738), XGBoost (AUC = 0.735), AdaBoost (AUC = 0.716), and Logistic Regression (AUC = 0.694). Random Forest also demonstrated the highest overall accuracy (0.697), specificity (0.691), F1 score (0.673), and AUPRC (0.701), indicating superior classification performance compared with the other models. In addition, Random Forest achieved the lowest Brier score (0.207), suggesting better calibration performance and more accurate probability estimation than the competing algorithms.

In the external validation cohort, GBM demonstrated the highest discrimination ability, with an AUC of 0.668 (95% CI: 0.614–0.721), followed by XGBoost (AUC = 0.654), Random Forest (AUC = 0.648), AdaBoost (AUC = 0.645), and Logistic Regression (AUC = 0.620). GBM also achieved the highest overall accuracy (0.648), specificity (0.679), AUPRC (0.554), and the lowest Brier score (0.238) among all evaluated models. Although predictive performance decreased in the external cohort compared with the internal cohort, all machine learning models demonstrated modest-to-moderate predictive performance.

Figures 3A,B present the ROC curves for the internal and external cohorts, respectively. Random Forest demonstrated the largest ROC area in the internal cohort, whereas GBM achieved the highest AUC in the external cohort. Calibration plots (Figures 3C,D) showed reasonable agreement between predicted and observed probabilities for the best-performing models in both cohorts. Precision-recall analyses (Figures 3E,F) further demonstrated model performance under class imbalance conditions. The highest AUPRC was achieved by Random Forest in the internal cohort (0.701) and by GBM in the external cohort (0.554), indicating superior precision-recall trade-offs compared with the other evaluated algorithms.

### 3.4 Model interpretation using SHAP

**FIGURE 4:**
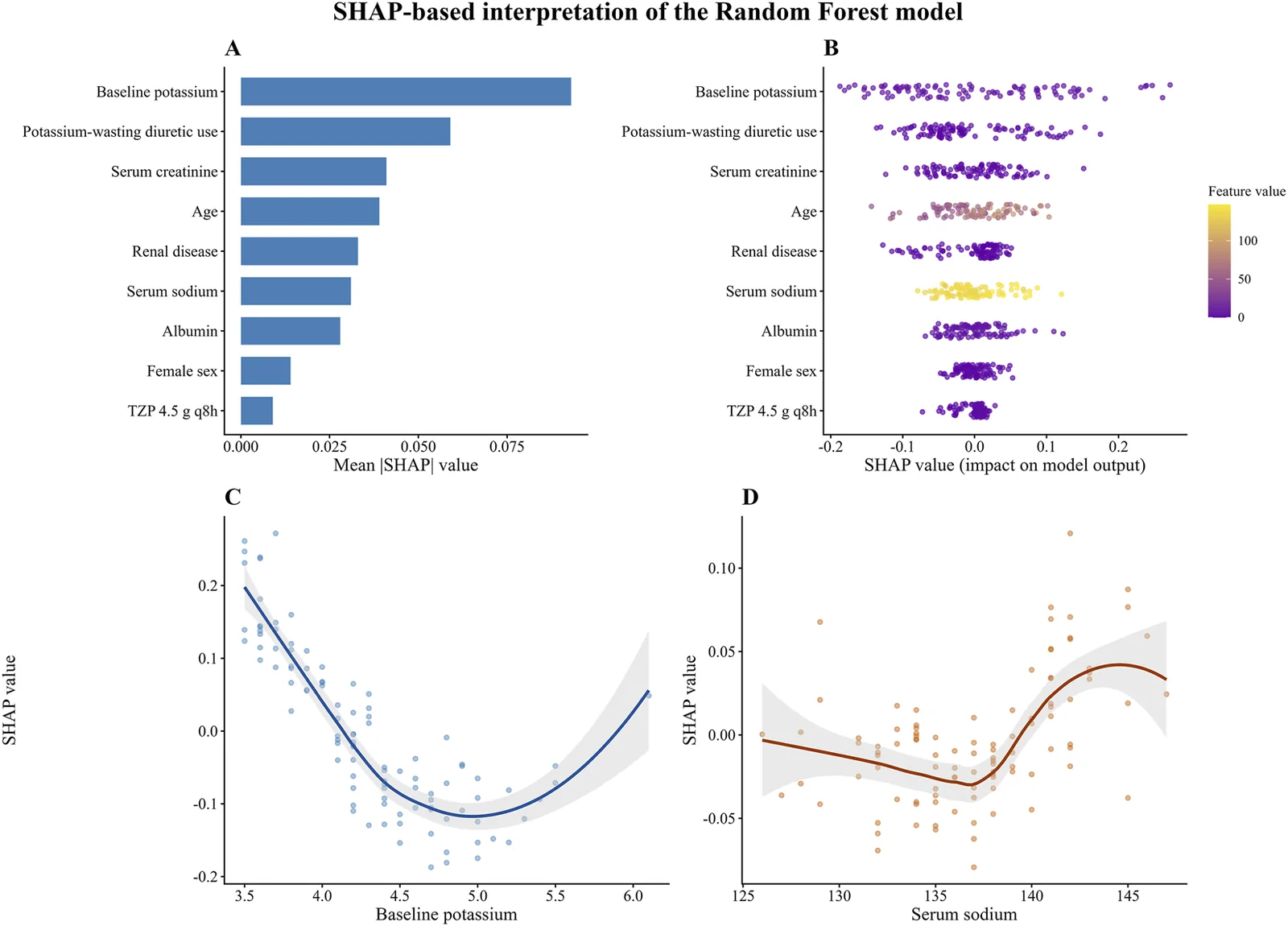
SHAP-based interpretation of the Random Forest model. **(A)** Feature importance ranked by mean absolute SHAP value. **(B)** SHAP summary plot. **(C)** SHAP dependence plot for baseline potassium. **(D)** SHAP dependence plot for serum sodium.

To improve the interpretability of the Random Forest model, SHAP analysis was performed (Table 3; Figure 4). Baseline potassium emerged as the most influential predictor, with a mean absolute SHAP value of 0.093. Lower baseline potassium levels were strongly associated with an increased predicted risk of hypokalemia. Potassium-wasting diuretic use was identified as the second most important predictor (mean |SHAP| = 0.059), highlighting the substantial contribution of medication-related potassium loss to the development of hypokalemia.

**TABLE 3:** SHAP-based predictor interpretation of the Random Forest model.

Variable	Mean |SHAP|	Clinical interpretation
Baseline potassium	0.093	Baseline potassium was the most influential predictor. Lower baseline potassium levels were strongly associated with an increased risk of hypokalemia
Potassium-wasting diuretic use	0.059	Concomitant use of potassium-wasting diuretics substantially increased susceptibility to potassium depletion and subsequent hypokalemia
Serum creatinine, mg/dL	0.041	Higher serum creatinine reflected impaired renal function and altered potassium handling, contributing to hypokalemia risk
Age, years	0.039	Advanced age was associated with increased vulnerability to electrolyte disturbances and hypokalemia
Renal disease	0.033	Renal disease contributed to model predictions through impaired electrolyte regulation and potassium homeostasis
Serum sodium, mmol/L	0.031	Serum sodium reflected electrolyte and volume-status related risk patterns associated with hypokalemia
Albumin, g/dL	0.028	Lower albumin levels reflected poorer nutritional and clinical status and were associated with increased hypokalemia risk
Female sex	0.014	Sex-related physiological differences exerted a modest influence on model predictions
TZP 4.5 g q8h	0.009	The TZP 4.5 g every 8 h regimen showed only a minor contribution to hypokalemia prediction

Abbreviations: SHAP, SHapley Additive exPlanations. Mean |SHAP| denotes the mean absolute SHAP, value and quantifies the average contribution of each predictor to the Random Forest model. Larger values indicate greater importance in predicting TZP-associated hypokalemia.

Other important predictors included serum creatinine (mean |SHAP| = 0.041), age (mean |SHAP| = 0.039), renal disease (mean |SHAP| = 0.033), and serum sodium (mean |SHAP| = 0.031). These variables collectively reflected the influence of renal function, electrolyte homeostasis, and patient-related susceptibility on hypokalemia risk. Albumin showed a moderate contribution (mean |SHAP| = 0.028), whereas female sex (mean |SHAP| = 0.014) and the TZP 4.5 g q8h regimen (mean |SHAP| = 0.009) demonstrated relatively small effects on model output.

The SHAP summary plot (Figure 4B) illustrated the distribution and direction of predictor effects across individual patients. Lower baseline potassium values consistently generated positive SHAP values, indicating an increased probability of hypokalemia, whereas higher baseline potassium values generally reduced model-predicted risk. The dependence plot for baseline potassium (Figure 4C) demonstrated a pronounced nonlinear relationship, with risk increasing substantially when baseline potassium levels fell below approximately 4.2 mmol/L.

Similarly, serum sodium exhibited a nonlinear association with model output (Figure 4D). Higher serum sodium concentrations tended to contribute positively to hypokalemia prediction, particularly at levels above approximately 140 mmol/L.

### 3.5 Risk-gradient analysis and decision curve analysis

**FIGURE 5:**
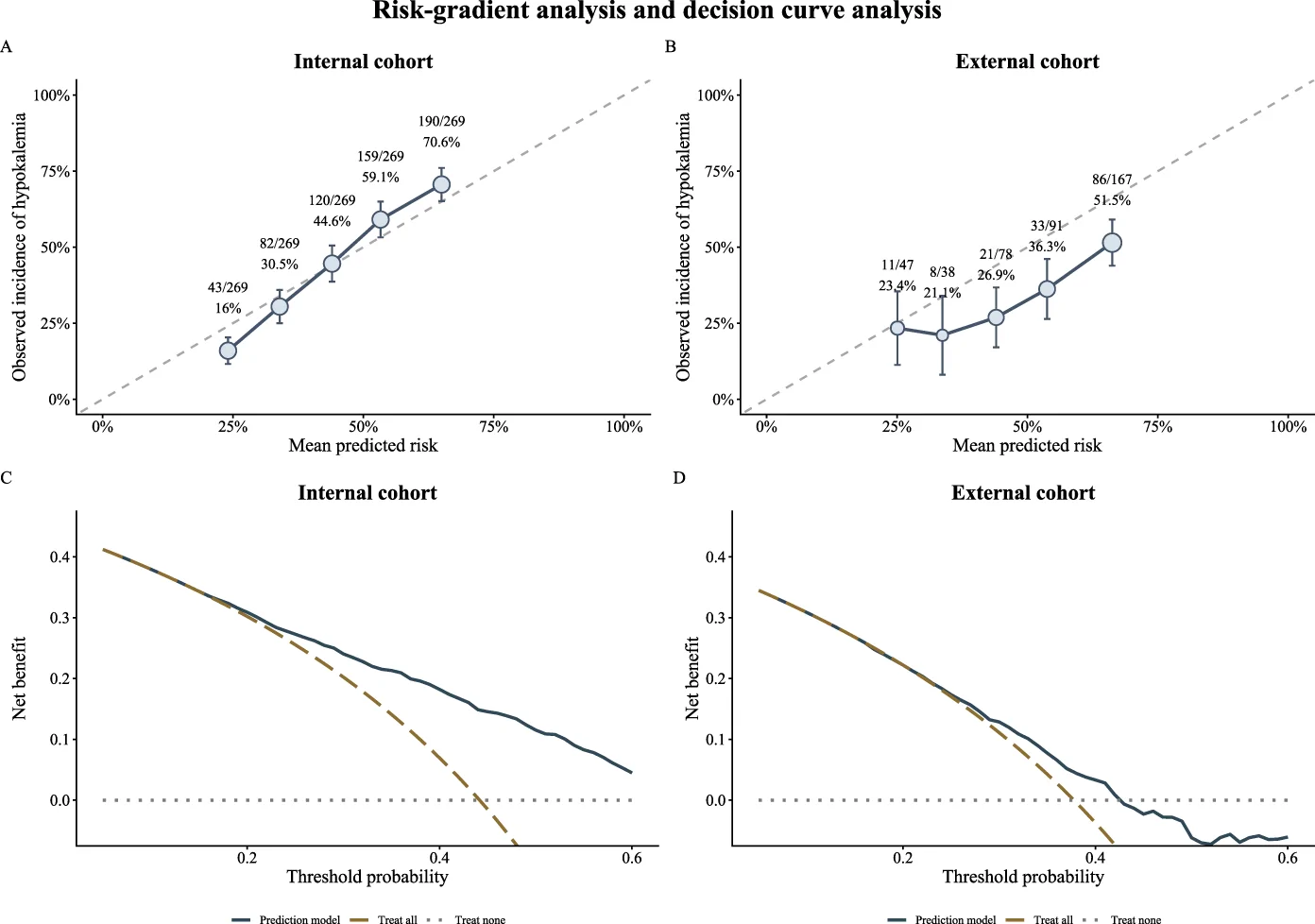
Risk-gradient analysis and decision curve analysis. **(A)** Internal risk-gradient analysis. **(B)** External risk-gradient analysis. **(C)** Internal decision curve analysis. **(D)** External decision curve analysis.

Risk-gradient analysis demonstrated a progressive increase in observed hypokalemia incidence across predicted risk strata in both the internal and external validation cohorts (Figures 5A,B). In the internal cohort, the observed incidence increased from 16.0% in the lowest-risk group to 70.6% in the highest-risk group. In the external validation cohort, the observed incidence increased from 23.4% to 51.5% across corresponding risk groups. The predicted and observed event rates showed reasonable agreement in the internal cohort, whereas wider confidence intervals and partial deviation from the reference line were observed in the external cohort.

Decision curve analysis demonstrated positive net clinical benefit across a range of threshold probabilities in the internal validation cohort (Figure 5C). The prediction model provided greater net benefit than the “treat-all” and “treat-none” strategies at threshold probabilities approximately between 0.15 and 0.60.

In the external validation cohort, the decision curve showed a narrower range of clinical benefit (Figure 5D). The prediction model maintained positive net benefit primarily within threshold probabilities between approximately 0.15 and 0.40.

## 4 Discussion

In this study, the incidence of TZP-associated hypokalemia was 44.16% (594/1,345) in the MIMIC-IV internal development cohort and 37.77% (159/421) in the external validation cohort from the Third Affiliated Hospital of Zunyi Medical University. Compared with previous reports, these incidences appear relatively high, likely reflecting the critically ill nature of the study population, prolonged hospitalization, frequent exposure to electrolyte-altering medications, and a higher burden of comorbidities. Most hypokalemia episodes were mild, whereas moderate and severe hypokalemia accounted for a smaller proportion of cases. Differences between cohorts may be attributable to variations in patient characteristics, disease severity, baseline electrolyte status, and clinical management practices (Cao et al., 2023; Lin et al., 2022).

Potassium is essential for maintaining membrane excitability, neuromuscular function, and cardiac electrophysiological stability. Hypokalemia has been associated with adverse clinical outcomes, including cardiac arrhythmias, muscle weakness, prolonged hospitalization, and increased mortality risk, particularly among critically ill patients (Seo and Kim, 2023; Timamy et al., 2024; Wan et al., 2026). Therefore, early identification of patients at risk of TZP-associated hypokalemia is clinically important and may facilitate timely intervention.

Among the evaluated machine learning algorithms, the Random Forest model demonstrated the best overall predictive performance, achieving an AUC of 0.759 in the internal cohort and 0.648 in the external validation cohort. Although model discrimination decreased during external validation, this finding is expected when models are applied across institutions with different patient populations and clinical settings. Nevertheless, calibration analysis demonstrated reasonable agreement between predicted and observed outcomes.

SHAP analysis further enhanced model interpretability and identified several clinically meaningful predictors. Baseline potassium emerged as the most influential predictor, with lower baseline potassium levels substantially increasing the predicted risk of hypokalemia. This finding is biologically plausible because patients with lower potassium reserves are more susceptible to further potassium depletion during TZP therapy. Potassium-wasting diuretic use was the second most important predictor, consistent with previous studies reporting an increased risk of TZP-associated hypokalemia among patients receiving concomitant potassium-depleting medications (Seo and Kim, 2022; Lin et al., 2022; Felker et al., 2020; Van der Heijden et al., 2019; Kuramoto et al., 2019).

Serum creatinine, age, renal disease, and serum sodium also contributed substantially to model predictions. These variables likely reflect the complex interactions between renal function, electrolyte homeostasis, and individual susceptibility to potassium disturbances. Renal disease and elevated serum creatinine may alter potassium handling through impaired tubular regulation and reduced physiological reserve, thereby increasing vulnerability to electrolyte imbalance. Similarly, serum sodium may serve as a marker of volume status and renal electrolyte regulation, both of which influence potassium homeostasis (Leegwater et al., 2022; Neuen et al., 2022; Sumida et al., 2022). Albumin demonstrated a moderate contribution to risk prediction, suggesting that nutritional status and overall clinical condition may also influence susceptibility to hypokalemia. Female sex and the TZP 4.5 g q8h regimen contributed less prominently but remained detectable predictors within the model.

Several biological mechanisms may explain the association between TZP exposure and hypokalemia. Piperacillin acts as a nonreabsorbable anion within the distal nephron, generating a lumen-negative electrical gradient that promotes renal potassium secretion (Seo and Kim, 2022; Kuramoto et al., 2019). In addition, the sodium load associated with intravenous TZP administration may increase distal sodium delivery and enhance urinary potassium excretion. These effects may be amplified in patients receiving potassium-wasting diuretics or in those with impaired renal function, thereby increasing the likelihood of hypokalemia (Lin et al., 2022; Felker et al., 2020; Van der Heijden et al., 2019).

The SHAP dependence plots further illustrated nonlinear relationships between predictor variables and model output. Lower baseline potassium concentrations were associated with a marked increase in predicted hypokalemia risk, particularly at concentrations below approximately 4.2 mmol/L. Serum sodium also demonstrated a nonlinear association with model predictions, indicating that electrolyte-related risk may not increase in a strictly linear manner. These findings support the biological plausibility of the model and highlight the multifactorial nature of TZP-associated hypokalemia.

From a clinical perspective, the present findings emphasize the importance of routine potassium monitoring in patients receiving TZP, especially among those with low baseline potassium levels, concomitant potassium-wasting diuretic exposure, renal dysfunction, or advanced age. Machine learning based risk stratification may provide supportive information for early identification of patients at higher risk of hypokalemia and may assist clinicians in optimizing monitoring strategies.

Several limitations should be acknowledged. First, this was a retrospective study and therefore remains susceptible to selection bias, residual confounding, and incomplete data capture. Second, although external validation was performed, both cohorts were derived from hospital-based populations, which may limit generalizability to other healthcare settings. Third, potentially important clinical variables, such as standardized severity scores, nutritional assessments, and detailed treatment information, were not consistently available and therefore could not be incorporated into model development. Finally, the decrease in predictive performance observed during external validation suggests that additional multicenter validation and prospective studies are required before routine clinical implementation. The use of a single imputed dataset may underestimate variability compared with full multiple imputation inference using Rubin’s rules.

## 5 Conclusion

We developed and externally validated a predictive model for piperacillin/tazobactam-associated hypokalemia in critically ill patients. Key clinical, laboratory, and treatment factors were identified, and the model demonstrated moderate discrimination and clinical utility, supporting early risk stratification and individualized potassium monitoring.

## Data Availability

The raw data supporting the conclusions of this article will be made available by the authors, without undue reservation.
